# Prognostics factors for mortality and renal recovery in critically ill patients with acute kidney injury and renal replacement therapy

**DOI:** 10.5935/0103-507X.20160015

**Published:** 2016

**Authors:** Sérgio Mina Gaião, André Amaral Gomes, José Artur Osório de Carvalho Paiva

**Affiliations:** 1 Department of Emergency and Intensive Care Medicine, Centro Hospitalar de São João, Faculdade de Medicina, Universidade do Porto - Porto, Portugal.; 2 Infection and Sepsis Group, Centro Hospitalar de São João, Faculdade de Medicina, Universidade do Porto - Porto, Portugal.

**Keywords:** Acute kidney injury, Renal insufficiency, Insufficiency renal, chronic, Renal replacement therapy, Intensive care

## Abstract

**Objective:**

Identify prognostic factors related to mortality and non-recovery of renal function.

**Methods:**

A prospective single-center study was conducted at the intensive care medicine department of a university hospital between 2012 and 2015. Patients with acute kidney injury receiving continuous renal replacement therapy were included in the study. Clinical and analytical parameters were collected, and the reasons for initiation and discontinuation of renal replacement therapy were examined.

**Results:**

A total of 41 patients were included in the study, of whom 43.9% had sepsis. The median Simplified Acute Physiology Score II (SAPSII) was 56 and the mortality was 53.7%, with a predicted mortality of 59.8%. The etiology of acute kidney injury was often multifactorial (56.1%). Survivors had lower cumulative fluid balance (median = 3,600mL, interquartile range [IQR] = 1,175 - 8,025) than non-survivors (median = 12,000mL, IQR = 6,625 - 17,875; p = 0.004). Patients who recovered renal function (median = 51.0, IQR = 45.8 - 56.2) had lower SAPS II than those who do not recover renal function (median = 73, IQR = 54 - 85; p = 0.005) as well as lower fluid balance (median = 3,850, IQR = 1,425 - 8,025 versus median = 11,500, IQR = 6,625 - 16,275; p = 0.004).

**Conclusions:**

SAPS II at admission and cumulative fluid balance during renal support therapy were risk factors for mortality and non-recovery of renal function among critically ill patients with acute kidney injury needing renal replacement therapy.

## Abstract (pt)

**Objetivo:**

Identificar fatores prognósticos relacionados com a mortalidade ou com a não recuperação da função renal.

**Métodos:**

Estudo monocêntrico, prospectivo, realizado em um serviço de medicina intensiva de um hospital universitário, entre 2012 e 2015. Incluíram-se doentes com lesão renal aguda em suporte renal contínuo. Foram coletados parâmetros clínicos e analíticos, assim como foi investigado o motivo para o início e o término do suporte renal.

**Resultados:**

Foram incluídos 41 doentes, 43,9% deles com sepse. O *Simplified Acute Physiology Score II* (SAPS-II) foi de 56, com mortalidade prevista de 59,8% e verificada de 53,7%. A etiologia da lesão renal aguda foi principalmente multifatorial (56,1%). Os sobreviventes apresentaram menor balanço hídrico acumulado (mediana de 3.600mL com intervalo interquartil de 1.175 - 8.025 *versus* 12.000mL [6.625 - 17.875] e p = 0,004. Os doentes que recuperaram função renal apresentaram SAPS II mais baixo do que os que não recuperaram (51,0 [45,8 - 56,2] *versus* 73 [54 - 85]; p = 0,005), assim como menor balanço hídrico (3850 [1.425 - 8.025] *versus* 11.500 [6.625 - 16.275]; p = 0,004).

**Conclusão:**

SAPS II na admissão e balanço hídrico acumulado durante o suporte renal foram fatores de risco para mortalidade e para a não recuperação da função renal em doentes graves com lesão renal aguda e necessidade de suporte renal.

## INTRODUCTION

The incidence of acute kidney injury (AKI) has increased considerably over the last two decades, particularly among inpatients.^(1-3)^ Currently, approximately 20% of critically ill patients experience at least one episode of AKI,^(4)^ 5% of whom requiring renal replacement therapy.^(5)^ The mortality rate of critically ill patients receiving renal replacement therapy because of AKI remains high, reaching approximately 50% cases, despite the growing understanding of the pathophysiology of AKI, the development of renal replacement therapy methods, the optimization of fluid resuscitation, and the choice of amine therapy.^(6)^ Incomplete renal function recovery is also common^(5)^ and has a significant effect on morbidity and mortality rates, quality of life, and healthcare costs.^(7)^

The prevalence of incomplete renal function recovery varies considerably across the studies published in the scientific literature.^(6,8)^ One possible explanation for this variation refers to the absence of a clear definition of renal function "recovery". Consequently, different definitions are used, and different prevalence rates are reported in turn. However, the possibility that certain therapeutic strategies, including the choice of renal replacement therapy, its start time, or the anticoagulant used, interfere with renal function recovery cannot be excluded.

The present study aimed to identify the prognostic factors related to mortality or renal function non-recovery in critically ill patients with AKI and renal replacement therapy.

## METHODS

A prospective single-center study was conducted in an intensive care unit (ICU) of a university hospital between 2012 and 2015. The Ethics Committee of *Centro Hospitalar São João* approved this study, and all participants or their family members signed an informed consent document. To be included in this study, the patients were between 18 and 90 years old, diagnosed with AKI, and were on continuous renal replacement therapy. The reasons for renal replacement therapy initiation were identified in a predefined multiple-choice table featuring items such as electrolyte disturbance, metabolic disorder, hypervolemia, oliguria/anuria, increased urea/creatinine, sepsis, and other, in which more than one option could be checked. The intensive care physicians made the decision to initiate continuous renal replacement therapy according to the standard of care practice, usually after considering the existence of hemodynamic instability requiring amine therapy, liver failure, or severe brain injury. The AKI classification stage was recorded using the Risk, Injury, Failure, Loss, and End-stage (RIFLE)^(9)^ criteria when renal replacement therapy was initiated. During the renal replacement therapy, patients were able to switch to intermittent renal replacement therapy according to the usual practices of the unit, usually after considering the absence of hemodynamic instability requiring amine therapy, liver failure, or severe brain injury.

The etiology of AKI was recorded based on the following options: sepsis, cardiogenic shock, hypovolemia, drug-induced nephrotoxicity, major surgery, use of contrast, obstructive uropathy, or other. More than one option could be selected. Patients were monitored based on the standard of care practice. Systolic and diastolic blood pressure, central venous pressure, urine output, use of diuretics, fluid balance, type of ventilation, use of amines, creatinine, urea, potassium, pH, and lactate were recorded from the initiation of renal replacement therapy to two days after its discontinuation. Records concerning the prescription of renal replacement therapy, including the type, dose and anticoagulant used were also performed.

The two major reasons justifying the discontinuation of renal replacement therapy were selected from a predefined multiple-choice table that included increased diuresis, improved metabolic/electrolyte status, improved hypervolemia, lowered urea/creatinine, hemodynamic stability, and other. The date of renal replacement therapy discontinuation was recorded, and its duration was calculated. The patient was allowed to receive continuous or intermittent therapy prior to discontinuation. The follow-up assessment of the patient was recorded at the ICU as well as another hospital department in the event that the patient had been transferred. We classified AKI non-recovery as: death during renal support therapy; death on RIFLE-F after discontinuation of renal replacement therapy; survivor continuing on renal replacement therapy; and survivor without renal replacement therapy but persistent RIFLE-F on hospital discharge. Acute kidney injury recovery was defined as a survivor without need for renal replacement therapy and without persistent RIFLE-F at hospital discharge. Non-survivors who died after renal replacement therapy and without RIFLE-F were also recorded as AKI recovery.

The continuous variables are expressed as percentages, medians, and interquartile ranges (IQR). Inter-group analyses were performed using the chi-square test and the Mann-WhitneyU test, where appropriate IBM Statistical Package for Social Science, version 20, was used for all analyses, and a 0.05 significance threshold was applied.

## RESULTS

The sample characteristics are outlined in table 1. Over two-thirds of the 41 patients had at least one comorbidity (i.e., high blood pressure, diabetes mellitus, heart failure, or cirrhosis). Eighteen patients (43.9%) had septic shock, and most of cases were related to medical disorders (73.2%). The median Simplified Acute Physiology Score II (SAPS II) was 56 (50 - 77), with a predicted mortality of 59.8%. Baseline creatinine was 1.0mg/dL (0.8 - 1.4), and the glomerular filtration rate estimated using the Modification of Diet in Renal Disease (MDRD) was 71mL/min (44 - 93), with 36.6% of patients showing glomerular filtration rates less than 60mL/min. The cumulative fluid balance during renal replacement therapy was 7,910mL.

**Table 1:** General data

**General data**		**General data**	
Number of patients	41	T0 creatinine (mg/dL)	2.8 (1.9 - 3.7)
Age (years)	67 (54 - 77)	T0 urea (mg/dL)	135 (88 - 159)
Men	28 (68.3)	T0 lactate (mmol/L)	3.1 (1.6 - 4.75)
SAPS II	56 (50 - 77)	T0 pH	7.3 (7.24 - 7.38)
Comorbidities		T0 hemoglobin (g/dL)	11.1 (9.4 - 12.1)
High blood pressure	19 (46.3)	T0 PaO_2_/FiO_2_	203 (140 - 235)
Heart failure	9 (22)	T0 albumin (g/L)	24 (19 - 26)
Diabetes mellitus	16 (39)	T0 central venous pressure (mmHg)	12 (10 - 14)
Cirrhosis	5 (12.2)	RIFLE	
Absent	12 (29.3)	R	5 (12.2)
Type of admission		I	17 (41.5)
Medical	30 (73.2)	F	19 (46.3)
Unscheduled surgery	11 (26.8)	Reason for initiating renal replacement therapy	
Baseline creatinine (mg/dL)	1.0 (0.8 - 1.4)	Electrolyte alterations	4 (9.8)
Glomerular filtration rate, MDRD (mL/min)	71 (44 - 93)	Metabolic alterations	24 (58.5)
Etiology o fAKI		Hypervolemia	8 (19.5)
Sepsis	18 (43.9)	Oliguria/anuria	22 (53.7)
Cardiorenal type I	9 (22)	Increased urea/creatinine	2 (4.9)
Hypovolemia	9 (22)	Sepsis	7 (17.1)
Pharmaceutical drugs	6 (14.6)	Other	4 (9.8)
Major surgery	7 (17.1)	Reason for discontinuing renal replacement therapy (among survivors)	
Contrast	10 (24.4)	Increased urine output	17 (81)
Urinary obstruction	1 (2.4)	Improved electrolyte/metabolic status	12 (57)
Other	10 (24.4)	Improved fluid status	3 (14)
Mechanical ventilation	37 (90.2)	Decreased urea/creatinine	0
Amine therapy	41 (100)	Hemodynamic stability	0 (0)

SAPS II - Simplified Acute Physiology Score II; MDRD - Modification of Diet in Renal Disease; T0 - initiation of renal replacement therapy; PaO_2_/FiO_2_ - partial pressure of oxygen/fraction of inspired oxygen ratio; RIFLE - Risk, Injury, Failure, Loss, End Stage. Results are expressed as medians (IQR) or rates (%).

The etiology of AKI was often multifactorial (56.1%), although sepsis was the predominant cause. All patients received amine therapy, and most received invasive mechanical ventilation at the initiation of renal replacement therapy (90.2%).

Approximately 61% of patients were admitted directly to the ICU, and the days of hospitalizations before ICU admission was 0 (0.0 - 4.0). The time between hospital admission and the initiation of renal replacement therapy was 2 days (0.5 - 7.5), and the time between admission to the ICU and the initiation of renal replacement therapy was 1 day (0 - 2). Continuous veno-venous hemofiltration was chosen to initiate renal replacement therapy for 87.8% of all cases, and continuous veno-venous hemodiafiltration was chose for 12.2% of all cases. In most cases, the prescription sought to ensure an effective dose of 20 - 25mL/kg/hour, except in cases of continuous veno-venous hemodiafiltration, given the temporary need for a high renal replacement therapy dose in the context of significant metabolic or electrolyte alterations, usually switching to continuous veno-venous hemofiltration during treatment. Twenty patients (48.8%) initiated renal replacement therapy without anticoagulation because of coagulation disorder, 15 (36.6%) were anticoagulated with heparin, and six (14.6%) received regional citrate anticoagulation. The solution buffer bicarbonate was exclusively used when regional citrate anticoagulation was not used. Seventeen patients (41.5%) died during renal replacement therapy. The median number of days on renal replacement therapy was 4.5 (1.2 - 7.8).

The length of hospital stay at the ICU was nine days (4 - 17.5). The ICU and hospital mortality rates were 48.8% and 53.7%, respectively.

No significant differences were observed between patients who survived and those who died in the ICU with regard to age, urea, creatinine at initiation of renal replacement therapy, creatinine at admission, glomerular filtration rate, serum albumin, hemoglobin, pH, PaO_2_/FiO_2_ ratio, need for amine therapy, number of days between hospital admission and initiation of renal replacement therapy, and number of days between ICU admission and initiation of renal replacement therapy (Table 2).

**Table 2:** Comparison between survivors and non-survivors in the intensive care unit

	**ICU survivors (N = 21)**	**ICU deceased (N = 20)**	**p-value**
Age (years)	64.0 (54.0 - 82.0)	67.5 (53.7 - 74.5)	0.651
Creatinine at admission (mg/dL)	1.0 (0.8 - 1.5)	1.0 (0.9 - 1.3)	0.860
Glomerular filtration rate, MDRD (mL/min)	68.0 (44.0 - 99.0)	71.5 (46.5 - 89.8)	0.938
T0 urea (mg/dL)	133 (82 - 149)	137 (89 - 168)	0.885
T0 creatinine (mg/dL)	2.9 (2.3 - 4.7)	2.1 (1.9 - 3.4)	0.420
T0 systolic blood pressure (mmHg)	93 (91 - 102)	95 (88 - 100)	0.885
T0 diastolic blood pressure (mmHg)	47 (41 - 51)	47 (43 - 53)	0.885
T0 central venous pressure (mmHg)	13 (10 - 14)	11.5 (9.3 - 14.8)	0.630
T0 albumin (g/L)	24 (21 - 27)	22 (17.3 - 25.8)	0.403
T0 lactate (mmol/L)	2.2 (1.5 - 4.8)	3.7 (2.0 - 4.8)	0.086
T0 Hb (g/dL)	11.1 (9.2 - 12.6)	10.7 (9.4 - 11.9)	0.885
T0 pH	7.31 (7.2 - 7.4)	7.29 (7.23-7.37)	0.873
RIFLE			
R	3 (14.3)	2 (10.0)	0.890
I	8 (38.1)	9 (45.0)	
F	10 (47.6)	9 (45.0)	
T0 PaO_2_/FiO_2_ ratio	210 (160 - 280)	192 (95 - 225)	0.425
Pre-ICU days	0 (0.0 - 1.0)	2.5 (0.0 - 9.75)	0.084
ICU stay (days)	14 (7.5 - 20.0)	5.5 (2.3 - 13.0)	0.158
Cumulative fluid balance (mL)	3600 (1175 - 8025)	12000 (6625 - 17875)	0.004
ICU/initiation of renal replacement therapy	1 (0.0 - 2.0)	1.0 (0.0 - 1.0)	0.541
Hospital admission/initiation of renal replacement therapy	1 (0.0 - 4.5)	3.5 (1.0 - 13.3)	0.276
SAPS II	51 (46 - 57)	77 (58 - 84)	0.005
Congestive heart failure	6	3	0.454
Cirrhosis	0	5	0.014

ICU - intensive care unit; MDRD - modification of diet in renal disease; T0 - initiation of renal replacement therapy; Hb - hemoglobin; RIFLE - Risk, Injury, Failure, Loss, End Stage; PaO_2_/FiO_2_ - partial pressure of oxygen/fraction of inspired oxygen ratio; SAPS II - Simplified Acute Physiology Score II. Results are expressed as medians (IQR) or rates (%).

Although survivors showed a lower level of lactacidemia (2.2mmoL/L [1.5 - 4.8] versus 3.7mmol/L (2.0 - 4.8]) and briefer hospital stays prior to admission to the ICU (zero days [0 - 1] versus 2.5 days (0 - 9.75]) than non-survivors, these results were not significant (p = 0.086 and p = 0.084, respectively).

Survivors showed lower SAPS II (51 [46 - 57] versus 77 (58-84]; p = 0.005) and lower cumulative fluid balance (3,600mL [1,175 - 8,025] versus 12.000mL (6,625 - 17,875]; p = 0.004) than the deceased. These differences were significant.

No differences were observed in age, creatinine at hospital admission, urea, creatinine, glomerular filtration rate, serum albumin, hemoglobin, pH, PaO_2_/FiO_2_ ratio at initiation of renal replacement therapy, need for amine therapy, and number of days between ICU admission and initiation of renal replacement therapy (Table 3).

**Table 3:** Comparison between patients with recovered renal function and those without recovered renal function

	**Recovered (N = 20)**	**Non-recovered (N = 21)**	**p-value**
Age (years)	62.0 (51.8 - 79.8)	67 (56.4 - 75.5)	0.860
Creatinine at admission (mg/dL)	1.0 (0.8 - 1.4)	1.0 (0.8 - 1.4)	0.860
Glomerular filtration rate, MDRD (mL/min)	69.5 (46.0 - 99.5)	72.0 (42.5 - 89.5)	0.835
T0 urea (mg/dL)	134 (78.5 - 146.0)	140 (90 - 166)	0.630
T0 creatinine (mg/dL)	2.8 (2.2 - 5.0)	2.1 (1.8 - 3.4)	0.650
T0 Systolic blood pressure (mmHg)	93 (91.5 - 103.2)	97 (89 - 100)	0.630
T0 Diastolic blood pressure (mmHg)	48.5 (42 - 54.2)	45 (42 - 50.0)	0.162
T0 Central venous pressure (mmHg)	12.5 (10.2 - 14.0)	12 (9.0 - 14.5)	0.885
T0 albumin (g/L)	24.5 (20.0 - 27.0)	24 (18.5 - 25.5)	0.278
T0 lactate (mmol/L)	2.5 (1.6 - 5.6)	3.5 (1.7 - 4.8)	0.873
T0 Hb (g/dL)	11.3 (10.2 - 12.4)	10.2 (8.6 - 12.0)	0.440
T0 pH	7.32 (7.18 - 7.38)	7.29 (7.24 - 7.38)	0.642
RIFLE			
R	3 (15.0)	2 (9.5)	0.406
I	9 (45.0)	8 (38.1)	
F	8 (40.0)	11 (52.4)	
T0 PaO_2_/FiO_2_ ratio	210 (160 - 280)	200 (95 - 225)	0.425
Pre-ICU days	0 (0 - 1.5)	2 (0.0 - 9.5)	0.140
ICU stay days	15 (8 - 25)	5 (2.5 - 12.5)	0.086
Cumulative fluid balance (mL)	3850 (1425 - 8025)	11500 (6625 - 16275)	0.004
ICU/initiation of renal replacement therapy	1.0 (0.0 - 2.0)	1.0 (0.0 - 1.5)	0.925
Hospital admission/initiation of renal replacement therapy	1.0 (0.0 - 4.0)	4.0 (1.0 - 15.0)	0.158
SAPS II	51 (45.8 - 56.2)	73 (54 - 84)	0.005
Congestive heart failure	4	5	0.77
Cirrhosis	0	5	0.020

MDRD - modification of diet in renal disease; T0 - initiation of renal replacement therapy; Hb - hemoglobin; RIFLE - Risk, Injury, Failure, Loss, End Stage; PaO_2_/FiO_2_ - partial pressure of oxygen/ fraction of inspired oxygen ratio; ICU - intensive care unit; SAPS II - Simplified Acute Physiology Score II. Results are expressed as medians (IQR) or rates (%).

Although patients with recovered renal function had fewer days between hospital admission and renal replacement therapy initiation as well as between hospital admission and ICU admission, these results were not significant (p = 0.158 and p = 0.14, respectively; Table 3).

The presence of cirrhosis was a risk factor for renal function non-recovery (p = 0.02). Patients with recovered renal function had lower SAPS II (51.0 [45.8 - 56.2] versus 73 [54 - 85]; p = 0.005) and lower cumulative fluid balance (3,850mL [1,425 - 8,025] versus 11,500mL [6,625 - 16,275]; p = 0.004) than those without recovered renal function (Table 3).

## DISCUSSION

SAPS II at admission, liver cirrhosis, and cumulative fluid balance during renal replacement therapy are risk factors for mortality and renal function non-recovery among critically ill patients with AKI needing renal replacement therapy.

The creatinine levels of our sample at ICU admission was 1.0mg/dL, which was slightly lower than that of other published studies.^(6,8)^ However, when the estimated glomerular filtration rate is included, the distribution of our results are identical to previous studies, such that 36.6% of our sample showed a glomerular filtration rate less than 60mL/min.

The creatinine and serum urea levels prior to renal replacement therapy were 2.8mg/dL and 135mg/dL, respectively, which are similar to those of other studies.^(6,8)^ However, the early renal replacement therapy initiation differs because more than 50% of the patients were in the R or I stages of the RIFLE criteria. Likewise, the number of days between hospital admission and the initiation of renal replacement therapy (2.0 [0.5 - 7.5]) was lower than that reported in the literature.^(6,8)^ Several reasons might explain this early renal replacement therapy. First, analytical alterations (urea/creatinine) were chosen as a criterion for initiation in only two cases (4.9%). Second, metabolic alterations (58.5%) and oliguria/anuria (53.7%), but not hypervolemia (19.5%), were the reasons given for initiating renal replacement therapy. Lastly, local factors explain why renal replacement therapy is initiated early at our unit.

Continuous veno-venous hemofiltration was the most common technique performed (87.8%). The other patients initially received continuous veno-venous hemodiafiltration, given the temporary need for a high renal replacement therapy dose in the context of significant metabolic or electrolyte alterations, usually switching to continuous veno-venous hemofiltration during treatment. Greater recovery of renal function after AKI has been observed among patients receiving continuous in place of intermittent renal replacement therapy;^(10-12)^ however, our study did not examine the use of the intermittent technique as an initial choice for renal replacement therapy.

Patients with AKI who discontinued renal replacement therapy but needed to resume it within seven days showed a higher mortality rate than those who successfully discontinued (seven consecutive days without requiring renal replacement therapy).^(13)^ Thus, accurate criteria of renal support discontinuation are crucial. However, few data exist concerning renal replacement discontinuation methods. Creatinine is clearly limited as an indicator for discontinuing of renal replacement therapy, being urine output the best predictor for it discontinuing, despite having a predictive value that is seriously affected by the use of diuretics.^(13,14)^ Our efficacy regarding discontinuing renal replacement therapy (i.e., only one patient required restarting the therapy because of a new renal insult resulting from hemorrhagic shock on the fifth day after discontinuing) might be explained by the fact that diuresis recovery was used as a stimulus to discontinue renal replacement therapy in most cases (81%), which is well above the 51% reported in a recently published survey conducted in the United States.^(15)^

In line with other studies,^(6,8,16)^ the hospital mortality rate of our population was high, reaching 53.7%. However, given that sepsis-induced AKI predicts high mortality rates,^(17)^ our populations had sepsis in 43.9% of cases, all received amine therapy and had a high need for mechanical ventilation (90.2%), the mortality was inferior to that predicted by SAPS-II.

Some differences were identified when comparing patients who died with those who survived. A higher level of lactacidemia as well as a longer period between hospital admission and ICU admission was noted among the deceased, matching previous reports.^(18)^ One possible explanation is that some patients might have been under-triaged to a hospital department other than the ICU or expressed refractoriness to a treatment already performed. In our study, however, neither outcome showed a significant difference. As expected, surviving patients had a lower SAPSII, with a lower mortality rate predicted from beginning. Several literature sources indicate that fluid accumulation in critically ill patients increases mortality, whereas only one study reported this finding with regard in renal replacement therapy.^(19)^ Our study confirmed this result, finding an association between positive fluid balance during renal replacement therapy and mortality among critically ill patients with AKI (p = 0.004).

Some studies have indicated that age, AKI intensity (urea, creatinine, or RIFLE classification), AKI etiology, or severity score are related factors in the context of AKI with regard to renal function recovery.^(20-23)^ However, those findings are not unanimous, and no strong relationship currently exists between those parameters and renal function recovery. Patients with recovered renal function in our sample had fewer days between hospital admission and renal replacement therapy initiation, although this result was not significant (p = 0.158), possibly because the sample was small. A recent meta-analysis clearly demonstrated this association, characterizing the early initiation of renal replacement therapy as a factor for improved renal function recovery.^(24)^

Patients with higher cumulative fluid balance during renal replacement therapy showed lower renal function recovery in a sub-analysis of the Randomized Evaluation of Normal versus Augmented Level Replacement Therapy (RENAL) trial that compared the doses of renal replacement therapy.^(19)^ Conversely, Silversides et al.^(25)^ did not find a relationship between fluid balance during renal replacement therapy and renal function recovery. However, this study only evaluated fluid balance over the first seven days after the initiation of renal replacement therapy and not throughout the entire period of renal replacement therapy. Our study was the first to addresses this topic as a primary outcome; in fact, our sample revealed that excess fluids accumulation during renal replacement therapy were associated with renal function non-recovery among patients with AKI requiring renal replacement therapy. This finding might be explained by increased venous pressure, intrarenal engorgement, and the subsequent decrease in the renal arteriovenous gradient, creating a sort of "renal compartment syndrome", as well as by the increased intra-abdominal pressure and consequent decrease of renal perfusion^(26)^ that leads to decreased renal function recovery capacity.

The present study has four major caveats. First, this study was observational and conducted at a single center; thus, the results are highly dependent on the standard of care of one department. Second, the sample size was small, which precludes the analysis of the results using a multivariate model. Third, a detailed description of the amines dosage was not performed throughout the renal replacement therapy; thus, we cannot exclude the possibility that the fluid balance resulted from greater hemodynamic instability. Fourth, only one patient had to resume renal replacement therapy after discontinuing.

Conversely, although some studies have identified risk factors for mortality in patients with AKI receiving renal replacement therapy, few studies thus far have researched the prognostic criteria for renal function recovery as a primary outcome.

## CONCLUSION

Critically ill patients with acute kidney injury who require renal replacement therapy have a high mortality rate, and the severity score at admission and the cumulative fluid balance during renal replacement therapy are poor prognostic factors. Some survivors are left with permanent kidney damage, witch accounts for an elevated morbidity and mortality at medium and long-term. An association was found between excessive fluid balance during renal replacement therapy and renal function non-recovery.

In fact, our study reported that critically ill patients with acute kidney injury receiving renal replacement therapy had one eventual modifiable risk factor to decrease their mortality rate and increase their renal function recovery, suggesting that volume management during renal replacement therapy could affects these variables.
